# Clinical Decision Making and Technical Approaches in Implantable Cardioverter-Defibrillator Procedures: A Step by Step Critical Appraisal of Literature

**DOI:** 10.31083/j.rcm2511403

**Published:** 2024-11-18

**Authors:** Eva Roseboom, Marcelle D. Smit, Hessel F. Groenveld, Michiel Rienstra, Alexander H. Maass

**Affiliations:** ^1^Department of Cardiology, University of Groningen, University Medical Center Groningen, 9713 GZ Groningen, The Netherlands; ^2^Department of Cardiology, Martini Hospital, 9728 NT Groningen, The Netherlands

**Keywords:** implantation, complications, perioperative, access

## Abstract

The selection of an appropriate implantable cardioverter-defibrillator (ICD) type and implantation strategy involves a myriad of considerations. While transvenous ICDs are standard, the rise of non-transvenous options like subcutaneous ICDs and extravascular ICDs is notable for their lower complication rates. Historical preferences for dual chamber ICDs have shifted to single-chamber ICDs. Single-coil ICDs are preferred for easier extraction, and the use of the DF-4 connector is generally recommended. Cephalic cutdown is the preferred venous access technique, while axillary vein puncture is a viable alternative. The right ventricular apex remains the preferred lead position until further evidence on conduction system pacing emerges. Left-sided, subcutaneous ICD implantation is considered reliable, contingent on specific cases. A meticulous perioperative plan, including antibiotic prophylaxis and an antithrombotic regimen, is crucial for successful implantation.

## 1. Introduction

**Fig. 1.**
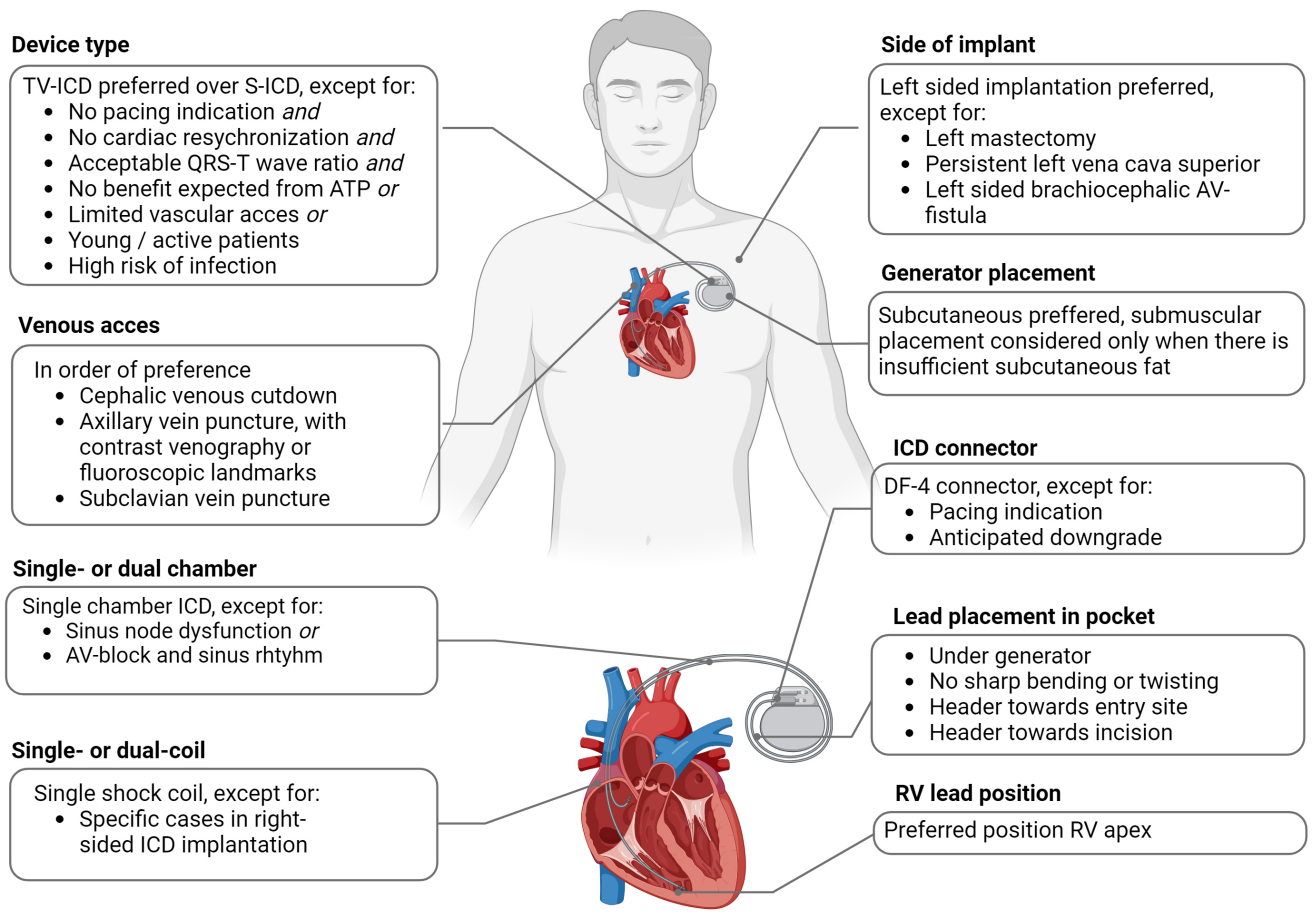
**Overview of recommendations for ICD implantation**. Abbreviations: ATP, anti-tachycardia pacing; AV-block, atrioventricular block; AV-fistula, arteriovenous fistula; ICD, implantable cardioverter-defibrillator; S-ICD, subcutaneous implantable cardioverter-defibrillator; TV-ICD, transvenous ICD; RV, right ventricular.

Implantation of an implantable cardioverter-defibrillator (ICD) has become a routine procedure, performed mostly by cardiologists and less by (cardio)thoracic surgeons. Experience and training significantly impact complication rates in ICD implantations [1, 2]. Many decisions have to be made in selecting the optimal implantation strategy, commencing with the selection of the device (single or dual-chamber ICD or cardiac resynchronization therapy-defibrillator (CRT-D)). Beyond device selection, strategic planning must extend to choices regarding venous access, determining the implantation side, selecting the appropriate connector type, and considering the lead positions. Given the heightened risks associated more complex device types and upgrade scenarios, a meticulously devised strategy becomes even more crucial [3]. In this review, we present our implantation strategies drawing from both literature and practical experience. Our aim is to provide the rationale behind each step in the ICD implantation pathway, as shown in Fig. 1.

## 2. ICD Devices and Lead Systems

### 2.1 Subcutaneous, Extravascular or Transvenous ICD Systems

Transvenous ICDs have proven their efficacy over the years, but the obligatory transvenous leads remain to be an Achilles’ heel, as they are responsible for most complications in ICD therapy. Pneumothorax, hemothorax, cardiac tamponade, and difficulties in achieving venous access are examples of short-term lead-related complications, while lead dislodgement, lead malfunction, venous occlusion, and device-related infection make up long-term complications attributed to transvenous leads [3].

The subcutaneous ICD (S-ICD) has been developed to overcome some of these complications, as it uses an extracardiac, subcutaneous electrode [4]. The implantation process for S-ICDs includes positioning the electrode along the thoracic wall and establishing a pocket in the upper left chest area. Opting for a subpectoral (or intermuscular) pocket — where the pulse generator is situated between the anterior surface of the serratus anterior and the posterior surface of the latissimus dorsi — may present several benefits over a purely subcutaneous pocket. Evidence from a prospective cohort study of 82 patients reveals that subpectoral placement is associated with enhanced first shock conversion efficacy for arrhythmias induced intra-operatively, fewer postoperative restrictions in left arm movement, a decreased incidence of inappropriate shocks, and a lower complication rate over an average follow-up duration of 3.6 ± 1.2 years. Additionally, the subpectoral approach is associated with better aesthetic outcomes and patient comfort relative to subcutaneous pulse generator placement [5]. The two-incision method for the electrode implantation, which omits the superior parasternal incision, has been suggested as a preferable alternative to the traditional three-incision technique. In a prospective cohort study of 39 patients, the two-incision approach was associated with a lower incidence of complications, fewer postoperative restrictions, and improved patient comfort compared to the three-incision method [6]. Therefore, this technique has been adopted by the implanting physicians at our hospital. Electrical signals are read at a distance from cardiac tissues, resulting in sensing values with lower amplitude, which are more susceptible to postural variation than endocardial leads [7, 8]. For proper discrimination, an adequate QRS amplitude and QRS to T-wave ratio is necessary. Defibrillation thresholds (DFT) are higher than in transvenous ICDs, and device size is larger to accommodate an 80 Joule charge. DFT is mandatory in S-ICD implantation [9]. An optimal implantation technique can reduce DFT and the PRAETORIAN score can be used to predict shock efficacy [10]. Even though obese patients have a higher risk of less successful termination of induced ventricular fibrillation (VF), selecting obese patients with a PRAETORIAN score of less than 90 can result in a good termination rate [11]. The S-ICD is unable to pace, except for optional, transient (30 seconds) post shock pacing, so it is unsuitable for patients with a pacing indication or known clinical indication for anti-tachycardia pacing (ATP). Median longevity of early generation S-ICD was 5 years but is expected to improve with the recent updates [12]. A study assessing premature battery depletion in S-ICDs potentially affected by a battery advisory found a 3.5% incidence of premature battery depletion after 4 years [13]. The shorter battery life is potentiated by much higher cost in most countries leading to cost per ICD therapy year that can increase 4–5-fold over transvenous ICD. This has to be taken into account if cost-effectiveness is an important factor in patient selection for implantation. To date, there have been two randomized clinical trials and multiple observational studies comparing transvenous ICD (TV-ICD) and S-ICD. The pivotal PRAETORIAN trial, published in 2020, serves as the cornerstone in this assessment, involving 849 patients, receiving an ICD primarily for primary prevention, randomly assigned in a 1:1 ratio [14]. Over a median follow-up period of 49 months, the trial found that the S-ICD exhibited noninferiority to TV-ICDs concerning the composite endpoint device-related complications and inappropriate shocks. Notably, S-ICDs were associated with more inappropriate shocks, whereas TV-ICDs had a higher incidence of device-related complications. Although S-ICD patients experienced more appropriate shocks, a substantial proportion of TV-ICD recipients benefited from ATP, mitigating shock frequency. The ATLAS trial is the first randomized controlled trial to have sought out superiority when comparing S-ICD to TV-ICD [15]. 544 patients were randomly assigned to receive either S-ICD of TV-ICD. Although underpowered to detect rates of failed or inappropriate shocks, they found a 92% decline in lead-related complications in the first 6 months after implantation. Further insights emerged from a retrospective cohort study involving 1160 patients, where propensity analysis on 280 individuals matched S-ICD and TV-ICD recipients [16]. This analysis revealed comparable complication rates but complication profiles differed. S-ICD recipients experienced fewer lead-related complications but more non-lead-related ones. Meanwhile, TV-ICD patients had a higher frequency of ICD interventions, primarily driven by ATP. The frequency of shocks, both appropriate and inappropriate, remained similar between the two groups. Additionally, the START trial focused on simulated sensing performance, demonstrating that both S-ICDs and TV-ICDs successfully detected all ventricular arrhythmias [17]. The European Society of Cardiology (ESC) guidelines for management of patients with ventricular arrhythmias state that the S-ICD should be considered as an alternative to TV-ICD in patients with an indication for an ICD when pacing therapy for bradycardia, cardiac resynchronization or ATP is not needed (class IIa level of evidence B) [18]. Ideal candidates for the S-ICD likely consist of young and highly active patients because of the anticipated longevity of the ICD lead, patients with limited vascular access, patients with high infection risk, and some groups of patients with congenital heart disease or hypertrophic cardiomyopathy, given the presence of acceptable QRS morphology and QRS to T-wave ratio [19]. Despite the availability of the S-ICD for several years in most European countries, its routine utilization remains limited, as demonstrated in a recent survey conducted by the European Heart Rhythm Association. Of the 52 centers that replied to this survey, 1/4th of centers did not implant S-ICDs. The most common indications for S-ICD implantation were limited vascular access (82%), history of previous complicated transvenous ICD (80%), young age (69%), and patients with an anticipated higher risk of infection (63%). 6% of centers suggested S-ICD as first choice therapy in patients without a pacing or CRT indication, and 18% of centers did not use a specific strategy to choose between transvenous ICD or S-ICD [20]. Real-world data from the international subcutaneous implantable cardioverter defibrillator registry highlight the specific selection of S-ICD recipients across patient populations compared to TV-ICD recipients. This study found that 58.1% of S-ICD recipients had nonischemic cardiomyopathies, with S-ICDs more commonly placed for primary prevention in patients with hypertrophic cardiomyopathy and Brugada syndrome [21]. There is increasing experience with the S-ICD for primary and secondary prevention in congenital heart disease, such as single ventricle physiology (including Fontan operation), transposition of the great arteries, and tetralogy of Fallot. The most common reasons for S-ICD implantation in such patients are limited transvenous access and intracardiac right-to-left shunt [22].

An emerging alternative to the TV-ICD is the extravascular ICD (EV ICD). In this implantation method, the shock lead is configured in an epsilon-shaped form to allow for passive fixation within the substernal space, and the arrangement of electrodes within the lead is optimized for effective sensing and pacing. The generator is subcutaneously positioned in the left midaxillary line, and is smaller than that of the S-ICD. In the EV ICD Pilot Study, a cohort of 21 patients underwent the implantation of the EV ICD. Successful implantation was achieved in 20 cases. Notably, 90% of these patients experienced successful termination of induced ventricular arrhythmias, while pacing capture was achieved in 95% of the patients. Importantly, there were no reported intraprocedural complications during the course of the study [23]. The Pivotal Study, a prospective, nonrandomized study involving 356 patients, 316 of whom underwent implantation, reported a successful defibrillation rate of 98.7% for induced ventricular arrhythmias. At six months, 92.6% of patients were free from major system- or procedure-related complications. However, during the mean follow-up period of 10.6 months, 29 patients experienced 118 inappropriate shocks, and eight systems were explanted [24]. Additionally, another study evaluated detection and sensing performance in 299 patients who were discharged with an EV ICD from the Pivotal Study. This evaluation found that the most common cause of oversensing was myopotentials (61.2%), followed by P-wave oversensing (19.9%) [25].

Based on the available literature in addition to the current guidelines, we prefer to implant S-ICDs in those patients who are expected to require ICD therapy for a long period and will therefore face repeat generator replacements. The risk of infection increases with each generator change, but the subcutaneous nature of the S-ICD reduces this risk compared to transvenous devices, which are more susceptible to severe infections. Additionally, S-ICDs are easier to extract than transvenous ICDs, minimizing procedural complexity. In these patients the more reliable subcutaneous lead of the S-ICD will, in the long-term, counterbalance the disadvantage of a slightly higher risk of inappropriate shocks. The EV ICD demonstrated reliable sensing and detection of VF, however, the frequency of inappropriate shocks was higher than that of the other implantation methods. Improvements in EV ICD technology and randomized trials are necessary to provide further recommendations.

### 2.2 Single- or Dual-Chamber ICD

One of the choices the ICD operator faces when implanting an ICD is the selection of a single- or dual-chamber device. If there is a pacing indication necessitating the implantation of a dual-chamber pacemaker system such as sinus node dysfunction or atrioventricular (AV)-block in sinus rhythm, a dual-chamber ICD (or VDD-system in selected patients, which uses a single lead for atrial sensing and ventricular pacing) is justified [9, 26]. However, the historical practice of implanting atrial leads in patients without pacing indications has been widespread. The reasoning behind this was that an additional atrial lead might enhance arrhythmia discrimination, potentially reducing the risk of inappropriate therapies. This assumption was supported by a meta-analysis which indeed showed a reduction in the number of inappropriate therapies in dual-chamber ICDs as compared with single-chamber ICDs, though the number of patients experiencing inappropriate therapies was not affected [27]. It is important to consider that these studies employed outdated programming methods, and contemporary heart failure management has substantially advanced since then. The annual rate of inappropriate shocks due to any cause has decreased significantly over the years, from 37–50% in the early days to 1–5% in the more recent studies [28, 29]. This reduction is likely due to advancements in ICD programming, primarily driven by longer detection intervals before delivering therapy or implementing high-rate therapy, where therapy is only delivered at high heart rates (e.g., >200 beats per minute) [30, 31]. This was further highlighted by a recent study comparing outcomes for primary prevention ICD patients at centers with high versus low adherence to 2015 and 2019 ICD programming guidelines, finding that patients at high guideline concordance centers experienced a significantly lower rate of ICD therapy, primarily due to reduced ATP therapy, without differences in mortality or first ICD shock rates [32].

Accordingly, most recent studies, consisting of randomized clinical trials and large registries mainly studying primary prevention patients, demonstrate that there is no difference in inappropriate therapies between single- and dual-chamber ICDs [28, 33]. Implantation of a dual-chamber device goes at the cost of increased periprocedural complications, in-hospital mortality, costs, and decreased battery life. Furthermore, accurate sensing of the atrial lead is of utmost importance when using atrial data to differentiate between supraventricular and ventricular tachycardia. Oversensing of far-field R-waves, undersensing in case of low-amplitude atrial signals, and atrial lead dislodgment can complicate arrhythmia discrimination. Hence, expert consensus and position papers have increasingly discouraged atrial lead implantation for the sole purpose of atrial arrhythmia detection or discrimination. The 2022 ESC guidelines now recommend single-chamber ICD implantation for patients without pacing indication [9].

### 2.3 Single- or Dual-Coil ICD Lead

Dual-coil leads, consisting of both a superior vena cava (SVC) defibrillation coil and a right ventricular coil, have conventionally been used to ensure the effective delivery of high-voltage shocks in ICDs. Nevertheless, these leads are associated with increased extraction challenges due to fibrosis often occurring around the proximal coil within the SVC. A comprehensive meta-analysis comprising 14 original studies that compared single-coil and dual-coil ICDs demonstrated equivalent first-shock efficacy and lower all-cause mortality among patients with single-coil ICDs [34]. In the context of right-sided ICDs, the consideration of a dual shock coil may be beneficial. A smaller study involving patients with right-sided ICDs, the inclusion of an SVC coil was found to reduce DFT in 45% of cases by modifying waveform duration [35]. DFT can, however, be even higher in selected patients when including an SVC coil. Therefore, when using a dual coil electrode, we advocate implanting an ICD capable of programming to include or exclude the SVC from shock paths.

### 2.4 ICD Lead Connector: DF-1 or DF-4

The ICD lead consists of a low-voltage pace-sense component and a high-voltage component used for defibrillation. The earliest ICDs had manufacturer-unique lead connectors that were only compatible with ICDs of the same manufacturer, creating the need for lead adapters and lead extenders in case of generator replacements [36]. Therefore, a standard DF-1 connector was developed. The pace-sense portion of the lead is connected to the ICD using a bipolar IS-1 lead connector which is inserted in the appropriate connector port in the ICD header, while the shock coil is inserted in another port in the ICD header using a DF-1 connector. An additional port for the second DF-1 connector is required in case of a dual-coil lead. The one or two high-voltage DF-1 connectors and the low-voltage IS-1 connector emerge from a bifurcated or trifurcated yoke at the proximal end of the lead. This arrangement, where the low- and high-voltage components leads are separated, adds to a considerable bulk of the ICD hardware. Manufacturers have therefore developed a new standard DF-4 connector, which incorporates high-voltage conductors together with the bipolar IS-1 pace-sense component into one connector pin [36, 37]. Benefits of the DF-4 connector includes a significantly smaller ICD header block, less connector bulk in the pocket, and simpler lead attachment to the ICD, eliminating the risk of incorrect device connection. Furthermore, lead dissection during generator replacement is much easier with less risk of lead damage. The first published experience with the DF-4 connector has been promising, with a trend towards a shorter procedure time, no difference in success of defibrillation threshold testing, stable lead measurements during follow-up, and no significant differences in lead failure as compared with DF-1 connectors [38]. However, the DF-4 connector also has disadvantages. It is less suitable for the management of high DFT, as additional hardware may be required to attain an acceptable defibrillation threshold [39]. In addition, there are concerns that, because the high- and low-voltage terminals reside on a single connector within a few millimeters of each other, high voltage can be electrically shorted to the low-voltage electrode. Noise from the shock electrode could also lead to potential oversensing on the low-voltage electrode [36]. Another important limitation is the inability to add a pace-sense lead in case of a malfunctioning pace-sense component of the ICD lead. Furthermore, the presence of a DF-4 lead connector makes it impossible to perform a downgrade to a pacemaker, which one could especially encounter in CRT-D patients in whom a downgrade to cardiac resynchronization therapy-pacemaker (CRT-P) is desired during follow-up. In these patients, the choice is left between generator replacement with another, expensive, CRT-D device in which the ICD function is turned off, or addition of a new right ventricular (RV) pace-sense lead to enable connection with a CRT-P device. This also applies to patients who might require future resynchronization by conduction system pacing (CSP) with left bundle branch area pacing or His bundle pacing in the event of CRT failure (non-responders). We therefore advocate the use of DF-1 lead connectors in ICD patients who also have a pacing indication and also in patients receiving a CRT-D, especially to be prepared for future downgrades which is increasingly performed in our institution [40].

## 3. Surgical Techniques and Approaches for ICD Implantation

### 3.1 Side of Implant

In the choice between left-sided and right-sided implantation for ICDs, studies have shown that left-sided ICDs tend to have more reliable outcomes. Prior research indicated varying results regarding right-sided ICDs, with higher defibrillation thresholds observed and reduced termination success of induced ventricular fibrillation with right-sided ICDs [41, 42, 43]. A European survey revealed that 79% of centers prefer left-sided placement for cardiac implantable electronic devices (CIEDs), while 10% favor the right side, and 11% consider patient handedness. Most operators (74%) do not modify implantation side based on device type. Given the evidence, we believe that left-sided implantation is desired in patients with an ICD [44, 45]. In a minority of patients exhibiting contraindications to left-sided implantation, such as cases involving left mastectomy, especially in the context of sentinel lymph node removal, persistent left superior vena cava, or left-sided arteriovenous fistula, a preference exists for right-sided implantation. For individuals engaging in rigorous physical activities that predominantly involve the left arm, the consideration of right-sided implantation or the preference for S-ICD implantation may arise. If right-sided implantation is inevitable, DFT testing should be considered to test reliability of the device [46].

### 3.2 Venous Access for Lead Implantation

Subclavian, axillary, and cephalic veins can all be used as venous access for lead implantation. Cephalic venous cutdown, preferred in 60% of centers according the European Heart Rhythm Association (EHRA) survey, typically begins with distal ligation followed by the option of either direct lead insertion or a sheath-assisted puncture [44]. It requires some surgical experience and the vein can be small or tortuous, which could make it difficult to insert one or more leads. In those situations, the utilization of guidewires and introducer sheaths can be helpful. The cephalic approach excludes most of the complications attributed to subclavian access, like pneumothorax and lead dysfunction risk, with a reported successful cannulation rate ranging from 60–80% and potentially exceeding 90% with hydrophilic guidewires or retro-pectoral veins [47, 48, 49]. Subclavian vein puncture, once popular because it is easy to learn, quick and carries a high success rate (approximately 95%), carries an important risk of complications such as pneumothorax (1–2%) and bleeding complications in the short-term, and subclavian crush syndrome in the long term, especially with ICD leads [3, 50, 51]. The puncture site should be as lateral as possible to avoid lead crush, and venography can help target the vein and mitigate risks. The puncture of the extra-thoracic subclavian vein or axillary vein has become increasingly popular due to its superior outcomes compared to the cephalic cutdown [52]. It also carries a reduced risk of lead failure compared to subclavian access, while presenting a similar bleeding risk. The axillary vein can be punctured using contrast venography, fluoroscopic landmarks or ultrasound. A large retrospective study compared the cephalic approach to subclavian puncture to assess perioperative complication rates. Among 139,176 pacemaker implantations, with 60.8% performed via the cephalic approach and 39.2% via subclavian access, the subclavian route demonstrated a higher complication rate compared to the cephalic approach (3.64% vs. 2.49%, respectively) [53]. A recently developed technique involves intra-pocket ultrasound-guided axillary vein puncture through the open incision using a small footprint probe. The ACCESS trial aimed to establish superiority in 200 patients undergoing CIED implantation [54]. Patients were randomly allocated to either intra-pocket ultrasound-guided axillary vein puncture or cephalic cutdown as the initial venous access approach. Intra-pocket ultrasound-guided axillary vein puncture demonstrated superiority in procedural success (defined as the successful insertion of all leads using the initially assigned venous access technique), as well as in time to achieve venous access, total procedure duration, fluoroscopy time, X-ray exposure, and complication rates. However, the fact that intra-pocket ultrasound is not employed universally across all centers may restrict the applicability of this comparison. When using the axillary vein, the incision often needs to be extended more laterally to allow space for lead fixation and to achieve a proper bend towards the pocket [55, 56]. Considering the above, it is recommended that subclavian access should be considered a bail-out technique and should be reserved for those situations where cephalic or axillary access is insufficient or unsuccessful.

In patients with previously implanted leads, significant lead-related venous obstruction can complicate device upgrade, lead revision, or lead replacement. In the last decade, two relatively large trials have reported a 27%–36% prevalence of significant stenosis as revealed by venography [57, 58]. Performing a selective venography prior to upgrade is considered useful to assess vessel patency and should preferably be performed sufficiently long before admission for the procedure, in order to be able to change strategy in case venous obstruction is observed. Caution is needed when using peripheral veins, for it may overestimate occlusion when the contrast dye spreads across the collaterals. Various approaches can mitigate venous obstruction. One method involves utilizing the contralateral side for lead and device implantation. Conversely, tunneling new leads is an option, albeit with an increased risk of superior vena cava syndrome. Alternatively, lead extraction can establish a conduit for new leads, but it entails a considerable risk of major complications and necessitates replacing functional leads [59]. Venoplasty, in which a percutaneous balloon angioplasty is carried out, creates a new entry for the placement of additional leads and is associated with a low perioperative and post-operative risk [60, 61]. When a transvenous option is not feasible, the alternatives of S-ICD or EV ICD should be considered.

### 3.3 Right Ventricular Lead Position

As per the 2013 EHRA survey, approximately 50% of the surveyed centers indicated the right ventricular apex (RVA) as their favored lead placement site. In contrast, about 47% preferred the interventricular septum, and only 3% favored the outflow tract [44]. The RVA has traditionally been preferred, owing to the ease of placement, low risk on lead dislodgment, and generally the favorable shocking vector. However, concerns have risen about possible detrimental effects of RV (apical) pacing in ICD patients, such as higher risk on development or worsening of heart failure [62, 63]. Though these adverse effects are more likely a consequence of the quantity of RV pacing instead of the pacing site in itself, several studies have explored the possible benefits and safety of other RV pacing sites, either in ICD patients or in CRT-D patients. On the whole, as compared with RV apex sites, non-apical RV sites such as RV outflow tract or RV septum show comparable results regarding defibrillation thresholds, appropriate or inappropriate therapies, lead function, or lead stability [64, 65, 66, 67]. In CRT-D patients, a non-apical RV lead position does not seem to be beneficial in terms of clinical outcome or echocardiographic response, and even has been associated with an increased risk of ventricular arrhythmias [68, 69]. Based on these results, apical placement of the RV lead should still be preferred. CSP is a novel cardiac pacing methodology, comprising His bundle pacing and left bundle branch area pacing, with the potential to restore physiological ventricular synchrony [70]. Notably, comprehensive trials assessing improvements in heart failure patient outcomes remain outstanding, however, it has already been incorporated into the latest HRS/APHRS/LAHRS guideline [71]. Consequently, this article doesn’t explore CSP pacing techniques, necessitating large-scale trials for long-term CSP versus biventricular pacing (BiVP) outcomes comparison [72].

### 3.4 Generator Placement Site

The first ICDs were bulky and heavy, which required abdominal implantation. As generator size decreased, pectoral implantation became more conventional. Submuscular implantation was favored in patients with a thin subcutaneous layer or was sometimes chosen for cosmetic reasons. In the early days of pectoral implantation of still quite bulky ICDs, short- and long-term complications and outcome variables were comparable between subcutaneous and submuscular implantation [73]. Generator size, however, has declined substantially over the years, and in our opinion the subcutaneous approach should nowadays be the method of choice. It is less painful and technically less demanding to create a subcutaneous pocket than a submuscular pocket. If desired, the generator can be secured to the muscle layer to reduce the risk of migration and, for example, Twiddler syndrome. Furthermore, generator replacement is much more challenging in patients with a submuscular pocket, which could lead to an increased risk of complications, in particular lead damage but also infection or pocket hematoma. In Europe, in accordance with our own views, the majority of operators indeed prefer a subcutaneous instead of a submuscular pectoral implantation of the ICD generator [44, 73].

### 3.5 Lead Placement in Pocket

**Fig. 2.**
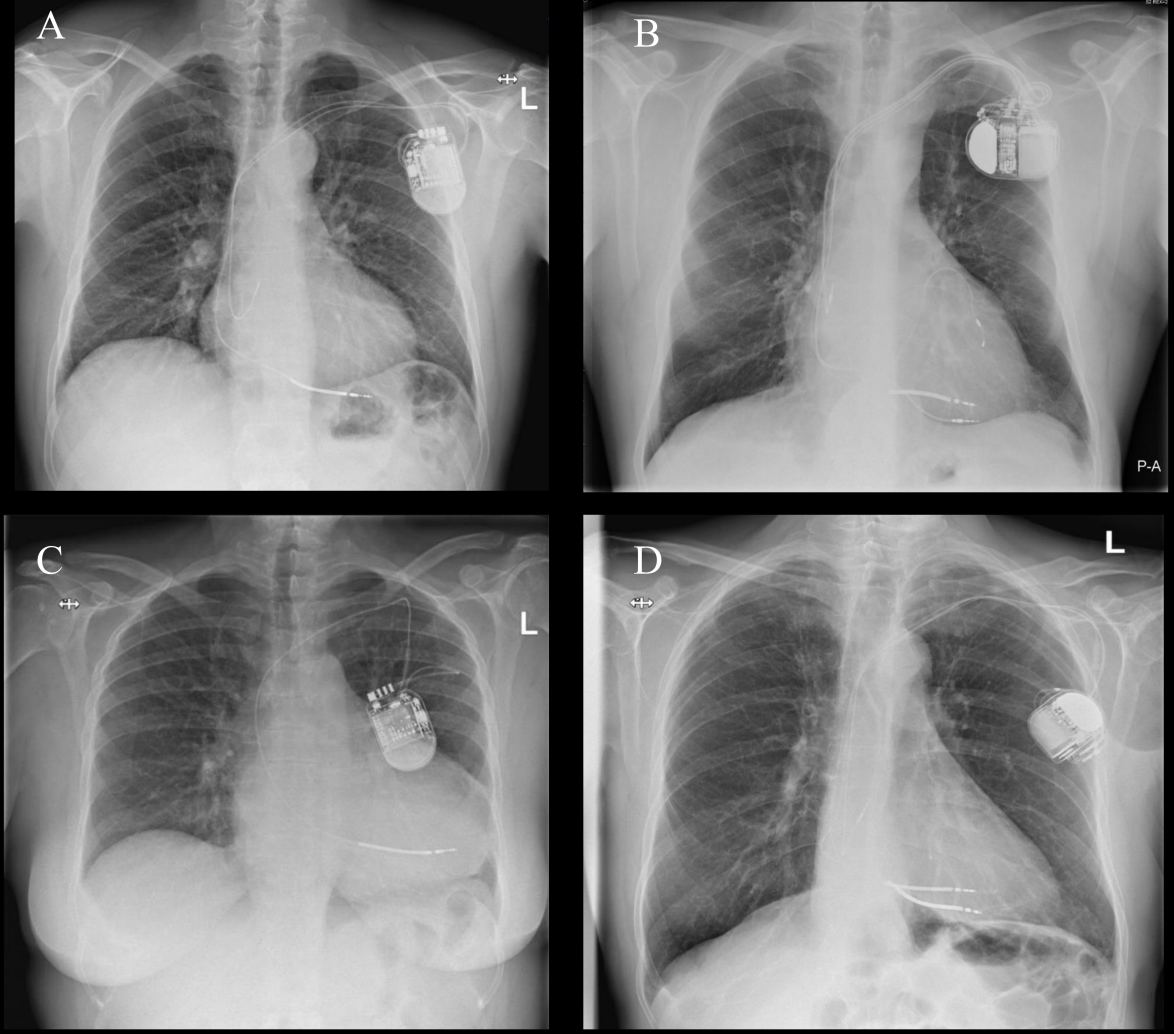
**Examples of optimal and suboptimal lead positioning in the pocket**. (A) Chest X-ray showing optimal placement of lead positioning in the pocket. (B) Chest X-ray showing suboptimal positioning of 180 ℃ twisting of leads near the header. (C) Chest X-ray showing suboptimal positioning of sharp bending at access site. (D) Chest X-ray showing suboptimal placement of header facing away from incision. P-A, posteroanterior; L, left.

In our opinion it is important to carefully place the leads in the pocket under the generator, taking care to avoid sharp bends and twists. This is crucial for the longevity of the leads. As we know from Twiddler syndrome, sharp bending and twisting of the leads can result in lead malfunctioning, lead failure, and inappropriate shocks [74]. To be able to store the excess lead in an optimal fashion, placing the device with the opening in the header towards the entry site in the vessel is preferable. Fig. 2 demonstrates examples of optimal and suboptimal lead positioning in the pocket. To prevent difficult device replacements, it is also crucial to point the header towards the incision.

## 4. Perioperative Measures

### 4.1 Antibiotic Prophylaxis

CIED-related infection occurs in 2–6% of patients and is a devastating complication, often requiring complete removal of the lead and generator system beside administration of antimicrobial therapy [75, 76, 77]. Mortality associated with device-related infection is reported to be up to 35% [78]. The majority of infections are caused by gram-positive staphylococci, predominantly *Staphylococcus aureus* [79]. Risk factors for infection can be categorized into patient-related and procedure-related factors. Patient-related factors include renal dysfunction, heart failure, diabetes, long-term corticosteroid use, and pre-procedural fever [80]. Procedure duration, procedure complexity (i.e., increasing number of leads, ICD versus pacemaker), inexperienced operator, omission of prophylactic antibiotics, and presence of postoperative hematoma are procedure-related factors associated with increased risk of infection [78, 80]. Incidence of infection is two- to five-fold higher in revision procedures. In line with increasing rates of left-sided endocarditis, the rate of primary device endocarditis without involvement of the pocket is increasing [81]. Antibiotic prophylaxis is essential to reduce device-related infection. As a primary choice, cefazolin is administered at a dose of 2 g or 3 g for patients weighing >120 kg. In situations where cephalosporin allergy or methicillin-resistant staphylococcal colonization is a concern, vancomycin (1 g) may be administered [19]. One randomized clinical trial, comparing 1 g cefazolin against placebo, administered immediately before primary device implantation or generator replacement, was terminated prematurely because of a significant difference in favor of the patients treated with antibiotics: 0.63% versus 3.28% of patients had local or systemic signs of infection [82].

Perioperative administration of antibiotics has become common practice, though not all issues concerning the implementation of antibiotic prophylaxis such as choice of antibiotic, timing, and optimal duration of therapy have been completely elucidated. Cefazolin seems to be first choice antibiotic agent, but studies directly comparing different antibiotics are lacking. Administering additional perioperative antibiotics does not yield additional advantages over conventional preoperative antibiotic therapy. The PADIT trial, involving 19,603 patients, found no significant difference in hospitalization rates for CIED infections within one year when comparing incremental treatment to standard preprocedural cefazolin [83]. The 2022 EHRA consensus document on how to prevent, diagnose, and treat CIED infections, recommends antibiotic prophylaxis within 1 hour of incision for cefazolin and flucloxacillin, and within 90–120 minutes for vancomycin [19]. This is based on the expectation that tissue and plasma antibiotic concentrations are maximal within one hour of intravenous administration, being optimal at time of incision and throughout the procedure. The optimal duration of therapy is also unknown, though repeat dosing of antibiotics does not seem to decrease risk of infection and is therefore not recommended after skin closure [83]. Neither local antiseptics on the wound and into the pocket, nor local antibiotic irrigation have so far been shown to decrease infection rates [83]. An antimicrobial mesh envelope releases minocycline and rifampin locally for ≥7 days, preventing CIED infections without extra complications. In the WRAP-IT trial (6983 patients), envelope recipients had fewer primary endpoints (infection requiring system revision, prolonged antibiotics, or death) within 12 months post-CIED implantation than controls (0.7% vs. 1.2%, HR 0.60; 95% CI 0.36–0.98; *p* = 0.04) [84]. However, the number needed to treat to prevent one infection was relatively high. Envelopes are likely to be the most cost-effective for patients at higher risk of infection, for example those undergoing lead upgrades or with a history of CIED infection. Individualized envelope use should consider risk factors and local CIED infection rates.

Other than antibiotic prophylaxis, certainly preventive measures such as a clean surgical environment, skin preparation and disinfection, hand antisepsis, good surgical technique, avoidance of temporary pacing, and prevention of pocket hematoma decrease risk of device infection [19]. While there is currently no data specific to CIED implantations, insights from hip surgery studies indicate a lower incidence of infections when employing chlorhexidine for skin disinfection in comparison to povidone-iodine. Consequently, we recommend the utilization of chlorhexidine, unless contraindicated due to a patient’s known allergy to this agent [85].

### 4.2 Antithrombotic Therapy

Pocket hematomas are an important complication of device implantation, as they can lead to prolonged interruption of antithrombotic therapy with subsequent increased risk of thromboembolism, increased hospitalization duration, need for hematoma evacuation, and increased risk of infection. A substantial proportion of patients undergoing device implantation receive one or multiple antithrombotic agents. Periprocedural management of antithrombotic therapy is challenging. The operator has to weigh up the risk of bleeding complications when continuing, against the risk of thromboembolic events when interrupting antithrombotic agents. This dilemma is most problematic in patients who carry a high risk for thromboembolic events. Regarding oral anticoagulation, the BRUISE CONTROL trial demonstrated that continuation of warfarin was associated with significantly less pocket hematomas than bridging with heparin (3.5% versus 16.0%) in patients with an annual risk of thromboembolic events of 5% or more [86]. This decreased risk was not influenced by type of device or type of implantation, i.e., primary implantation versus generator change or upgrade. Median international normalized ratio (INR) was 2.3 (interquartile range 2.0–2.6) in the continued warfarin group and 1.2 (interquartile range 1.1–1.3) in the heparin bridging group. Thromboembolic complications were rare and did not differ between the groups. Other studies also consistently demonstrated a significantly higher risk of bleeding events with heparin bridging compared to continuation of oral anticoagulation, with no significant difference in thromboembolic events [87, 88]. With regard to other types of antithrombotic agents, bleeding events may be lowest with antiplatelet therapy, especially aspirin, as compared with oral anticoagulants and novel oral anticoagulants (NOACs) [88]. Concerns regarding increased bleeding risk with one or more combinations of antithrombotic agents were discarded in an observational study, demonstrating no significant differences in pocket hematomas between patients with no antithrombotic therapy, with oral anticoagulant therapy, with single or dual antiplatelet therapy, or with combinations of oral anticoagulants and one or two antiplatelets [89]. Instead of type and number of antithrombotic agents, high HAS-BLED score and presence of valvular disease were independent predictors of significant pocket hematoma. NOACs may be associated with higher risk of bleeding events than antiplatelets [88]. The BRUISE CONTROL-2 trial investigated the effects of continued versus interrupted NOACs on clinically significant hematoma in patients with moderate to high risk on thromboembolic events [90]. The trial was terminated early due to futility; event rates were much lower than expected, and continuation of NOAC was not superior to interruption of NOAC. Considering the currently available evidence, we interrupt oral anticoagulants and NOACs perioperatively in patients carrying a low risk on thromboembolic events. Aspirin is continued, and clopidogrel or ticagrelor are continued in case of drug-eluting stent implantation <1 year. In patients carrying a high risk of thromboembolic events, e.g., with a mechanical valve, electrical cardioversion <4 weeks, or CHA2DS2-VASc score of 8–9, oral anticoagulation is continued. In the rare context of NOAC use in the mentioned high-risk situations, implantation can occur safely with uninterrupted NOAC use [90].

### 4.3 Defibrillation Threshold Testing

For many years, defibrillation efficacy has been tested by DFT, especially to establish appropriate connection of the ICD lead and to test whether the ICD is adequately able to detect and terminate VF with a shock. Technology and programming have improved over the years, leading to reduced energy requirements for defibrillation. Furthermore, DFT is not without risk and can even result in death [26]. Possible complications of DFT include myocardial injury, contractile dysfunction leading to worsening heart failure, central nervous system injury due to hypoperfusion, thromboembolic events in the presence of intracardiac thrombus or atrial fibrillation, or respiratory depression due to anesthetic drugs. Such complications have resulted in a decline in perioperative DFTs. Several studies examining DFT testing at ICD implantation with current-generation devices have consistently shown no significant differences in outcomes [91, 92, 93]. These trials, like the SIMPLE and NORDIC ICD trials, have indicated that not performing DFT testing is noninferior to DFT testing, with a trend towards superiority in terms of fewer adverse events [94, 95]. A systematic review and meta-analysis further support the absence of significant differences in mortality or adverse outcomes between patients with and without DFT testing [96]. In the absence of randomized data or society guidelines, many electrophysiologists still opt for DFT testing during S-ICD implantation, particularly for S-ICD generator replacement procedures. However, observational data have not demonstrated a lower rate of ineffective shocks or cardiovascular mortality associated with DFT at initial S-ICD implantation [97, 98]. Considering these results, the HRS/EHRA/APHRS/SOLAECE consensus statement on optimal ICD programming states that it is reasonable to omit DFT in patients undergoing left pectoral transvenous ICD implantation where appropriate sensing (>5–7 mV), pacing, and impedance values are obtained with fluoroscopically well-positioned RV leads (class IIa recommendation) [26]. On the contrary, patients receiving a non-transvenous subcutaneous ICD should undergo DFT (class I recommendation). Patients underrepresented in the studies mentioned above include those with hypertrophic obstructive cardiomyopathies or channelopathies, those undergoing generator replacement, and those with right-sided ICD implantation. In such patients’ performance of DFT is reasonable (class IIa recommendation). In our part of the country where *phospholamban* gene mutation-associated cardiomyopathy is prevalent, performance of DFT should also be advocated because of the low-voltage electrocardiograms in these patients.

## 5. Other Challenges in ICD Implantation

### 5.1 Aberrant Anatomy 

When implanting any CIED, we want to be prepared for possible obstacles. We always check patients’ history and anatomy to be aware of possible aberrant venous anatomy such as persistent left superior vena cava, presence of arteriovenous fistulas in hemodialysis patients, and presence of venous obstruction and collaterals caused by, e.g., radiotherapy or lymph node dissection. We conduct an echocardiogram in every patient, as a persistent left superior vena cava can be recognized. We consider contrast venography if venous access may be difficult such in patients with previous central venous catheter with longer dwell times, radiation therapy, or thoracic surgery. Persistence of left superior vena cava is present in 0.2–0.4% of patients undergoing CIED implantation [99]. As discussed previously, left-sided implantation of an ICD is preferable but this can be more difficult in the presence of a persistent left superior vena cava. Right superior vena cava is missing in 30% of patients with left superior vena cava so right-sided implantation is not always a bailout option in these patients. Studies have shown that careful implantation with appropriate stylet shaping and the use of active fixation leads can result in successful transvenous implantation with reliable long-term device performance in most cases [100, 101]. CIEDs are implanted in 0.7% of hemodialysis patients [102]. Presence of arteriovenous fistulas or the necessity to create one is an important obstacle in these patients. Arteriovenous fistula failure is frequently observed in patients with CIEDs, especially for fistulas that are located ipsilateral to the device [103]. Indeed, CIEDs are associated with high rates, i.e., >60%, of central vein stenosis in hemodialysis patients, of which half of patients have to sacrifice their fistula and become catheter dependent for their hemodialysis afterwards [102]. We therefore try to avoid implantation of ICDs and pacemakers ipsilateral to the arteriovenous fistula. If there is no other option, contrast venography is conducted to check fistula anatomy as fistulas are sometimes connected to the cephalic vein. If conventional transvenous lead implantation is impossible due to anatomic obstacles, implantation of epicardial pacing leads or defibrillator coils through minimally invasive surgical approaches are a possibility with low associated morbidity [104]. Otherwise, implantation of an S-ICD can be considered, as previously discussed in the section on S-ICDs.

### 5.2 Strategy for Upgrades/Revisions

Device upgrades and revisions entail the implantation of one or more additional leads. Upgrades and revisions are the most difficult implantation procedures and are associated with the highest complication rates, so it is essential to be thoroughly prepared. As mentioned before, venography should be performed to assess vessel patency. If patients with a pacemaker require an upgrade to an ICD, our advice is to use the old pace/sense lead as it has proved to be reliable in these patients, who are frequently pacemaker dependent, and avoid a DF-4 lead connector.

## 6. Conclusions

The evolving landscape of ICD technologies underscores the importance of a comprehensive approach, blending historical considerations with contemporary innovations, and emphasizing the significance of a meticulous perioperative plan to ensure the success of implantation procedures. We provided a rationale in navigating the complexities of ICD selection and implantation strategy.
